# Machine learning and serving of discrete field theories

**DOI:** 10.1038/s41598-020-76301-0

**Published:** 2020-11-09

**Authors:** Hong Qin

**Affiliations:** Plasma Physics Laboratory, Princeton University, Princeton, NJ 08543 USA

**Keywords:** Information theory and computation, Scientific data

## Abstract

A method for machine learning and serving of discrete field theories in physics is developed. The learning algorithm trains a discrete field theory from a set of observational data on a spacetime lattice, and the serving algorithm uses the learned discrete field theory to predict new observations of the field for new boundary and initial conditions. The approach of learning discrete field theories overcomes the difficulties associated with learning continuous theories by artificial intelligence. The serving algorithm of discrete field theories belongs to the family of structure-preserving geometric algorithms, which have been proven to be superior to the conventional algorithms based on discretization of differential equations. The effectiveness of the method and algorithms developed is demonstrated using the examples of nonlinear oscillations and the Kepler problem. In particular, the learning algorithm learns a discrete field theory from a set of data of planetary orbits similar to what Kepler inherited from Tycho Brahe in 1601, and the serving algorithm correctly predicts other planetary orbits, including parabolic and hyperbolic escaping orbits, of the solar system without learning or knowing Newton’s laws of motion and universal gravitation. The proposed algorithms are expected to be applicable when the effects of special relativity and general relativity are important.

## Introduction and statement of the problem

Data-driven methodology has attracted much attention recently in the physics community. This is not surprising since one of the fundamental objectives of physics is to deduce or discover the laws of physics from observational data. The rapid development of artificial intelligence technology begs the question of whether such deductions or discoveries can be carried out algorithmically by computers.

In this paper, I propose a method for machine learning of discrete field theories in physics from observational data. The method also includes an effective algorithm to serve the discrete field theories learned, in terms of predicting new observations.

Machine learning is not exactly a new concept in physics. In particular, the connection between artificial neural networks and dynamical systems has been noticed for decades^1–8^. What is the new contribution brought by the present study? Most current applications of machine learning techniques in physics roughly fall into the following categories. (i) Using neural networks to model complex physical processes, such as plasma disruptions in magnetic fusion devices^9–12^, effective Reynolds stress due to turbulence^13^, coarse-grained nonlinear effects^14^, and proper moment closure schemes for fluid systems^15^. (ii) Solving differential equations in mathematical physics by approximating solutions with neural networks^16–21^. In particular, significant progress has been made in solving Schrödinger’s equation for many-body systems^22,23^. (iii) Discovering unknown functions or undetermined parameters in governing differential equations^24–33^. As a specific example, methods of learning the Hamiltonian function of a canonical symplectic Hamiltonian system were proposed very recently^34–41^. (iv) Using neural networks to generate sampling data in statistical ensembles for calculating equilibrium properties of physical systems^42–45^.

The problem addressed in this paper belongs to a new category. The method proposed learns a field theory from a given set of training data consisting of observed values of a physical field at discrete spacetime locations. The laws of physics are fundamentally expressed in the form of field theories instead of differential equations. It is thus more important to learn the underpinning field theories when possible. Since field theories are in general simpler than the corresponding differential equations, learning field theories is easier, which is true for both human intelligence and artificial intelligence. Except for the fundamental assumption that the observational data are governed by field theories, the learning and serving algorithms proposed do not assume any knowledge of the laws of physics, such as Newton’s law of motion and Schrödinger’s equation. This is different from most existing methodologies of machine learning in physics.

Without loss of generality, let’s briefly review the basics of field theories using the example of first-order field theory in the space of ${\text {R}}^{n}$ for a scalar field $\psi$. A field theory is specified by a Lagrangian density $L(\psi ,\partial \psi /\partial x^{\alpha }),$ where $x^{\alpha }$ $(\alpha =1,...,n)$ are the coordinates for $\mathrm {R}^{n}$. The theory requires that with the value of $\psi$ fixed at the boundary, $\psi (x)$ varies with respect to *x* in such a way that the action of the system

1 $$\begin{aligned} {\mathcal {A}}=\int L(\psi ,\partial \psi /\partial x^{\alpha })d^{n}x \end{aligned}$$

is minimized. Such a requirement of minimization is equivalent to the condition that the following Euler-Lagrange (EL) equation is satisfied everywhere in $\mathrm {R}^{n},$,

2 $$\begin{aligned} EL(\psi )\equiv \sum _{\alpha =1}^{n}\frac{\partial }{\partial x^{\alpha }}\left( \frac{\partial L}{\partial \left( \partial \psi /\partial x^{\alpha }\right) }\right) -\frac{\partial L}{\partial \psi }=0\,. \end{aligned}$$

The problem of machine learning of field theories can be stated as follows:

**Problem Statement 1**. For a given set of observed values of $\psi$ on a set of discrete points in $\mathrm {R}^{n},$ find the Lagrangian density $L(\psi ,\partial \psi /\partial x^{\alpha })$ as a function of $\psi$ and $\partial \psi /\partial x^{\alpha }$, and design an algorithm to predict new observations of $\psi$ from *L*.

Now it is clear that learning the Lagrangian density $L(\psi ,\partial \psi /\partial x^{\alpha })$ is easier than learning the EL equation (2), which depends on $\psi$ in a more complicated manner than *L* does. For example, the EL equation depends on second-order derivatives $\partial ^{2}\psi /\partial x^{\alpha }\partial x^{\beta }$ and *L* does not. However, learning *L* from a given set of observed values of $\psi$ is not an easy task either for two reasons. Suppose that *L* is modeled by a neural network. We need to train *L* using the EL equation, which requires the knowledge of $\partial ^{2}\psi /\partial x^{\alpha }\partial x^{\beta }.$ For this purpose, we can set up another neural network for $\psi (x)$, which needs to be trained simultaneously with *L*. This is obviously a complicated situation. Alternatively, one may wish to calculate $\partial ^{2}\psi /\partial x^{\alpha }\partial x^{\beta }$ from the training data. But it may not be possible to calculate them with desired accuracy, depending on the nature of the training data. Secondly, even if the optimized neural network for *L* is known, serving the learned field theory by solving the EL equation with a new set of boundary conditions presents a new challenge. The first-order derivatives $\partial \psi /\partial x^{\alpha }$ and second-order derivatives $\partial ^{2}\psi /\partial x^{\alpha }\partial x^{\beta }$ are hidden inside the neural network for *L*, which is nonlinear and possibly deep. Solving differential equations defined by neural networks ventures into uncharted territory.

As will be shown in “Machine learning and serving of discrete field theories” section, reformulating the problem in terms of discrete field theory overcomes both difficulties. Problem Statement 1 will be replaced by Problem Statement 2 in “Machine learning and serving of discrete field theories” section. To learn a discrete field theory, it suffices to learn a discrete Lagrangian density $L_{d}$, a function with $n+1$ inputs, which are the values of $\psi$ at $n+1$ adjacent spacetime locations. The training of $L_{d}$ is straightforward. Learning serves the purpose of serving, and the most effective way to serve a field theory with long term accuracy and fidelity is by offering the discrete version of the theory, as has been proven by the recent advances in structure-preserving geometric algorithms^46–75^. Therefore, learning a discrete field theory directly from the training data and then serving it constitute an attractive approach for discovering physical models by artificial intelligence.

It has long been theorized since Euclid’s study on mirrors and optics that as the most fundamental law of physics, all nature does is to minimize certain actions^76,77^. But how does nature do that? The machine learning and serving algorithms of discrete field theories proposed might provide a clue, when incorporating the basic concept of the simulation hypothesis by Bostrom^78^. The simulation hypothesis states that the physical universe is a computer simulation, and it is being carefully examined by physicists as a possible reality^79–81^. If the hypothesis is true, then the spacetime is necessarily discrete. So are the field theories in physics. It is then reasonable, at least from a theoretical point of view, to suggest that some machine learning and serving algorithms of discrete field theories are what the discrete universe, i.e., the computer simulation, runs to minimize the actions.

In “Machine learning and serving of discrete field theories” section, the learning and serving algorithms of discrete field theories are developed. Two examples of learning and predicting nonlinear oscillations in 1D are given in “Examples of learning and predicting nonlinear oscillations” section to demonstrate the method and algorithms. In “Kepler problem” section, I apply the methodology to the Kepler problem. The learning algorithm learns a discrete field theory from a set of observational data for orbits of the Mercury, Venus, Earth, Mars, Ceres, and Jupiter, and the serving algorithm correctly predicts other planetary orbits, including the parabolic and hyperbolic escaping orbits, of the solar system. It is worthwhile to emphasize that the serving and learning algorithms do not know, learn, or use Newton’s laws of motion and universal gravitation. The discrete field theory directly connect the observational data and new predictions. Newton’s laws are not needed.

## Machine learning and serving of discrete field theories

In this section, I describe first the formalism of discrete field theory on a spacetime lattice, and then the algorithm for learning discrete field theories from training data and the serving algorithm to predict new observations using the learned discrete field theories. The connection between the serving algorithm and structure-preserving geometric integration methods is highlighted.

To simplify the presentation and without losing generality, the theory and algorithms are given for the example of a first-order scalar field theory in $\mathrm {R}^{2}$. One of the dimension will be referred to as time with coordinate *t*, and the other dimension space with coordinate *x*. Generalizations to high-order theories and to tensor fields or spinor fields are straightforward.

**Figure 1:**
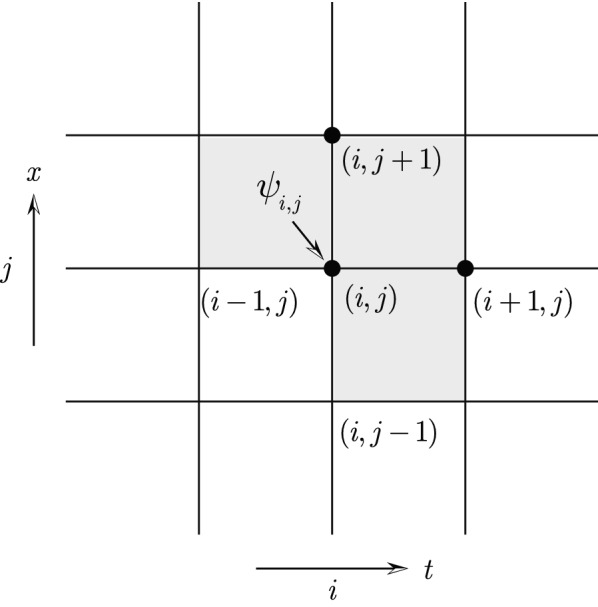
Spacetime lattice and discrete field $\psi$. The discrete Lagrangian density $L_{d}(\psi _{i,j},\psi _{i+1,j},\psi _{i,j+1})$ of the grid cell whose lower left vertex is at the grid point (*i*, *j*) is chosen to be a function of the values of the discrete field at the three vertices marked by solid circles. The action ${{\mathcal {A}}}_{d}$ of the system depends on $\psi _{i,j}$ through the discrete Lagrangian densities of the three neighboring grid cells indicated by gray shading.

For a discrete field theory in $\mathrm {R}^{2}$, the field $\psi _{i,j}$ is defined on a spacetime lattice labeled by two integer indices (*i*, *j*). For simplicity, let’s adopt a rectangular lattice shown in Fig. 1. The first index *i* identifies temporal grid points, and the second index *j* spacial grid points. The discrete action ${{\mathcal {A}}}_{d}$ of the system is the summation of discrete Lagrangian densities over all grid cells,

3 $$\begin{aligned} {{\mathcal {A}}}_{d}=\Delta t\Delta x\sum _{i,j}L_{d}(\psi _{i,j},\psi _{i+1,j},\psi _{i,j+1})\,, \end{aligned}$$

where $\Delta t$ and $\Delta x$ are the grid sizes in time and space respectively, and $L_{d}(\psi _{i,j},\psi _{i+1,j},\psi _{i,j+1})$ is the discrete Lagrangian density of the grid cell whose lower left vertex is at the grid point (*i*, *j*). I have chosen $L_{d}$ to be a function of $\psi _{i,j}$, $\psi _{i+1,j}$, and $\psi _{i,j+1}$ only, which is suitable for first-order field theories. For instance, in the continuous theory for wave dynamics, the Lagrangian density is

4 $$\begin{aligned} L\left( \psi ,\frac{\partial \psi }{\partial t},\frac{\partial \psi }{\partial x}\right) =\left( \frac{\partial \psi }{\partial t}\right) ^{2}-\left( \frac{\partial \psi }{\partial x}\right) ^{2}\,. \end{aligned}$$

Its counterpart in the discrete theory can be written as

5 $$\begin{aligned} L_{d}(\psi _{i,j},\psi _{i+1,j},\psi _{i,j+1})=\left( \frac{\psi _{i+1,j}-\psi _{i,j}}{\Delta t}\right) ^{2}-\left( \frac{\psi _{i,j+1}-\psi _{i,j}}{\Delta x}\right) ^{2}\,. \end{aligned}$$

The discrete Lagrangian density $L_{d}$ defined in Eq. (5) can be viewed as an approximation of the continuous Lagrangian density *L* in Eq. (4). But I prefer to take $L_{d}$ as an independent object that defines a discrete field theory.

For the discrete field theory, the condition of minimizing the discrete action ${{\mathcal {A}}}_{d}$ with respect to each $\psi _{i,j}$ demands

6 $$\begin{aligned} EL_{i,j}(\psi )&\equiv \frac{\partial {{\mathcal {A}}}_{d}}{\partial \psi _{i,j}}=\frac{\partial }{\partial \psi _{i,j}}\left[ L_{d}(\psi _{i-1,j},\psi _{i,j},\psi _{i-1,j+1})\right. \nonumber \\&\quad \left. +L_{d}(\psi _{i,j},\psi _{i+1,j},\psi _{i,j+1})+L_{d}(\psi _{i,j-1},\psi _{i+1,j-1},\psi _{i,j})\right] =0\,. \end{aligned}$$

Equation (6) is called Discrete Euler-Lagrange (DEL) equation for the obvious reason that its continuous counterpart is the EL equation (2) with $x^{1}=t$ and $x^{2}=x$. Following the notation of the continuous theory, I also denote the left-hand-side of the last equal sign in Eq. (6) by an operator $EL_{i,j}(\psi ),$ which maps the discrete field $\psi _{i,j}$ into another discrete field. The DEL equation is employed to solve for the discrete field $\psi$ on the spacetime lattice when a discrete Lagrangian density $L_{d}$ is prescribed. This has been the only usage of the DEL equation in the literature that I am aware of so far^48,49,51–53,55,57,58,61,64,68,70–75^. I will come back to this shortly.

For the problem posed in the present study, the discrete Lagrangian density $L_{d}$ is unknown. It needs to be learned from the training data. Specifically, in terms of the discrete field theory, the learning problem discussed in “Introduction and statement of the problem” section becomes:

**Problem Statement 2**. For a given set of observed data ${\overline{\psi }}_{i,j}$ on a spacetime lattice, find the discrete Lagrangian density $L_{d}(\psi _{i,j},\psi _{i+1,j},\psi _{i,j+1})$ as a function of $\psi _{i,j}$, $\psi _{i+1,j}$, and $\psi _{i,j+1}$, and design an algorithm to predict new observations of $\psi _{i,j}$ from $L_{d}$.

Unlike the difficult situation described in “Introduction and statement of the problem” section for learning a continuous field theory, learning a discrete field theory is straightforward. The algorithm is obvious once the problem is declared as in Problem Statement 2. We set up a function approximation for $L_{d}$ with three inputs and one output using a neural network or any other approximation scheme adequate for the problem under investigation. The approximation is optimized by adjusting its free parameters to minimize the loss function

7 $$\begin{aligned} F({\overline{\psi }})=\frac{1}{IJ}\sum _{i=1}^{I-1}\sum _{j=1}^{J-1}EL_{i,j}({\overline{\psi }})^{2} \end{aligned}$$

on the training data ${\bar{\psi }}$, where *I* and *J* are the total number of grid points in time and space respectively. In Problem Statement 2 and the definition of loss function (7), it is implicitly assumed that the training data are available over the entire spacetime lattice. Notice that according to Eqs. (6) and (7), first-order derivatives of $L_{d}$ with respect to all three arguments are required to evaluate $F({\overline{\psi }})$. Automatic differential algorithms^29^, which have been widely adopted in artificial neural networks, can be applied. To train the neural network or other approximation for $L_{d},$ established methods, including Newton’s root searching algorithm and the Adam optimizer^82^, are available.

Once the discrete Lagrangian density $L_{d}$ is trained, the learned discrete field theory is ready to be served to predict new observations. After boundary conditions are specified, the DEL equation (6) is solved for the discrete field $\psi _{i,j}$. A first-order field theory requires two boundary conditions in each dimension. As an illustrative example, let’s assume that $\psi _{0,j}$ and $\psi _{1,j}$ are specified for all *j*s, corresponding to two initial conditions at $t=0,$ and $\psi _{i,0}$ and $\psi _{i,1}$ are specified for all *i*s, corresponding to two boundary conditions at $x=0.$ Under these boundary and initial conditions, the DEL equation (6) can be solved for field $\psi _{i,j}$ for all *i*s and *j*s as follows.

Step  1: Start from the DEL equation at $(i,j)=(1,2),$ i.e., $EL_{1,2}(\psi )=0,$ which is an algebraic equation containing only one unknown $\psi _{2,2}.$ Solve $EL_{1,2}(\psi )=0$ for $\psi _{2,2}$ using a root searching algorithm, e.g., Newton’s algorithm.
Step  2: Move to grid point $(i,j)=(1,3).$ Solve the DEL equation $EL_{1,3}(\psi )=0$ for the only unknown $\psi _{2,3}.$
Step  3: Repeat Step 2) with increasing value of *j* to generate solution $\psi _{2,j}$ for all *j*s.
Step  4: Increase index *i* to 2. Apply the same procedure in Step 3) for generating $\psi _{2,j}$ to generate $\psi _{3,j}$ for all *j*s.
Step  5: Repeat Step 4) for $i=3,4,...,I$ to solve for all $\psi _{i,j}$.

In a nutshell, the DEL equation at the grid cell labeled by (*i*, *j*) (see Fig. 1) is solved as an algebraic equation for $\psi _{i+1,j}$. This serving algorithm propagates the solution from the initial and boundary conditions to the entire spacetime lattice. It is exactly how the physical field propagates physically. According to the simulation hypothesis, the algorithmic propagation and the physical propagation are actually the same thing. When different types of boundary and initial conditions are imposed, the algorithm needs to be modified accordingly. But the basic strategy remains the same. Specific cases will be addressed in future study.

The above algorithms in $\mathrm {R}^{2}$ can be straightforwardly generalized to $\mathrm {R}^{n},$ where the discrete Lagrangian density $L_{d}$ will be a function of $n+1$ variables, i.e, $\psi _{i_{1},i_{2},...,i_{n}}$, $\psi _{i_{1}+1,i_{2},...,i_{n}}$, $\psi _{i_{1},i_{2}+1,...,i_{n}}$,......, $\psi _{i_{1},i_{2},...,i_{n}+1}$. And in a similar way as in $\mathrm {R}^{2}$, the serving algorithm solves for $\psi _{i_{1},i_{2},...,i_{n}}$by propagating its values at the boundaries to the entire lattice. It can also be easily generalized to vector fields or spinor fields, as exemplified in “Kepler problem” section.

It turns out this algorithm to serve the learned discrete field theory is a variational integrator. The principle of variational integrators is to discretize the action and Lagrangian density instead of the associated EL equations. Methods and techniques of variational integrators have been systematically developed in the past decade^46–75^. The advantages of variational integrators over standard integration schemes based on discretization of differential equations have been demonstrated. For example, variational integrators in general are symplectic or multi-symplectic^48,49,51–53,55,57,58,61,64,68,70–75^, and as such are able to bound globally errors on energy and other invariants of the system for all simulation time-steps. More sophisticated discrete field theories have been designed to preserve other geometric structures of physical systems, such as the gauge symmetry^52,75^ and Poincaré symmetry^72,80,81,83^. What proposed in this paper is to learn the discrete field theory directly from observational data and then serve the learned discrete field theory to predict new observations.

## Examples of learning and predicting nonlinear oscillations

In this section, I use two examples of learning and predicting nonlinear oscillations in 1D to demonstrate the effectiveness of the learning and serving algorithms. In 1D, the discrete action reduces to the summation of the discrete Lagrangian density $L_{d}$ over the time grids,

8 $$\begin{aligned} {{\mathcal {A}}}_{d}=\Delta t\sum _{i=0}^{I}L_{d}(\psi _{i},\psi _{i+1})\,. \end{aligned}$$

Here, $L_{d}(\psi _{i},\psi _{i+1})$ is a function of the field at two adjacent time grid points. The DEL equation is simplified to

9 $$\begin{aligned} EL_{i}(\psi )\equiv \frac{\partial L_{d}(\psi _{i-1},\psi _{i})}{\partial \psi _{i}}+\frac{\partial L_{d}(\psi _{i},\psi _{i+1})}{\partial \psi _{i}}=0\,. \end{aligned}$$

The training data ${\bar{\psi }}_{i}$ $(i=0,...,I)$ form a time sequence, and the loss function on a data set $\psi$ is

$$\begin{aligned} F(\psi )=\frac{1}{I}\sum _{i=1}^{I-1}EL_{i}(\psi )^{2}\,, \end{aligned}$$

After learning $L_{d}$, the serving algorithm will predict a new time sequence for every two initial conditions $\psi _{0}$ and $\psi _{1}$. Note that Eq. (9) is an algebraic equation for $\psi _{i+1}$ when $\psi _{i-1}$ and $\psi _{i}$ are known. It is an implicit two-step algorithm from the viewpoint of numerical methods for ordinary differential equations. It can be proven^49,51^ that the algorithm exactly preserves a symplectic structure defined by

10 $$\begin{aligned}&\Omega (\psi _{i},\psi _{i+1})=d\theta \,, \end{aligned}$$

11 $$\begin{aligned}&\theta =\frac{\partial L_{d}(\psi _{i},\psi _{i+1})}{\partial \psi _{i+1}}d\psi _{i+1}\,. \end{aligned}$$

The algorithm is thus a symplectic integrator, which is able to bound globally the numerical error on energy for all simulation time-steps. Compared with standard integrators which do not possess structure-preserving properties, such as the Runge-Kutta method, variational integrators deliver much improved long-term accuracy and fidelity.

For each of the two examples, the training data taken by the learning algorithm are a discrete time sequence generated by solving the EL equation of an exact continuous Lagrangian. In 1D, the EL equation is an Ordinary Differential Equation (ODE) in time. Only the training sequence is visible to the learning and serving algorithms, and the EL equation and the continuous Lagrangian are not. After learning the discrete Lagrangian from the training data, the algorithm serves it by predicting new dynamic sequences $\psi _{i}$ for different initial conditions. The predictions are compared with accurate numerical solutions of the EL equation.

Before presenting the numerical results, I briefly describe how the algorithms are implemented. To learn $L_{d}(\psi _{i},\psi _{i+1})$, a neural network can be set up. Since it has only two inputs and one output, a deep network may not be necessary. For these two specific examples, the functional approximation for $L_{d}(\psi _{i},\psi _{i+1})$ is implemented using polynomials in terms of $s\equiv \psi _{i}+\psi _{i+1}$ and $d\equiv \psi _{i+1}-\psi _{i}$, i.e.,

12 $$\begin{aligned} L_{d}(\psi _{i},\psi _{i+1})=\sum _{p=0}^{P}\sum _{q=0}^{Q}a_{pq}d^{p}s^{q}\,, \end{aligned}$$

where $a_{pq}$ are trainable parameters. For these two examples, I choose $(P,Q)=(4,8)$, and the total number of trainable parameters are 45. For high-dimensional or vector discrete field theories, such as the Kepler problem in “Kepler problem” section, deep neural networks are probably more effective.

### *Example 1*

**Figure 2:**
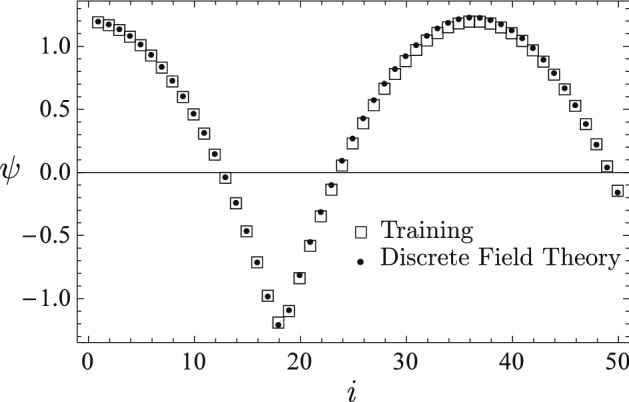
The predicted sequence $\psi _{i}$ (solid circles) from the learned discrete field theory and the training sequence ${\bar{\psi }}_{i}$ (empty squares) are barely distinguishable in the figure. The discrete Lagrangian is trained until the loss function $F({\overline{\psi }})$ is less than $10^{-7}$.

The training data are plotted in Fig. 2 using empty square markers. It is a time sequence ${\bar{\psi }}_{i}$ $(i=0,...,50)$ generated by the nonlinear ODE

13 $$\begin{aligned} 2\left( \sin \psi +1\right) \psi ^{\prime \prime }+\left( \psi ^{\prime }\right) ^{2}\cos \psi +\frac{\pi ^{2}}{200}\psi =0 \end{aligned}$$

with initial conditions $\psi (t=0)=1.2$ and $\psi ^{\prime }(t=0)=0.$ Here $\psi ^{\prime }$ denote $d\psi /dt$. The Lagrangian density for the system is

14 $$\begin{aligned} L(\psi ,\psi ^{\prime })=\left( 1+\sin \psi \right) \left( \psi ^{\prime }\right) ^{2}-\frac{\pi ^{2}}{400}\psi ^{2}\,. \end{aligned}$$

The optimizer for training the discrete Lagrangian density $L_{d}$ is Newton’s algorithm with step-lengths reduced according to the amplitude of loss function. The discrete Lagrangian density $L_{d}$ is trained until the loss function on ${\bar{\psi }}$ is less than $10^{-7}$, then it is served. Plotted in Fig. 2 using solid circle markers are the predicted time sequence $\psi _{i}$ using the initial conditions of the training data, i.e., $\psi _{0}={\bar{\psi }}_{0}$ and $\psi _{1}={\bar{\psi }}_{1}.$ The predicted sequence $\psi _{i}$ and the training sequence ${\bar{\psi }}_{i}$ are barely distinguishable in the figure.

**Figure 3:**
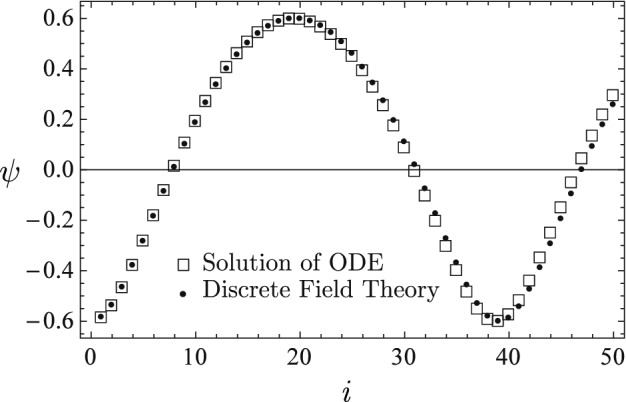
The predicted time sequence (solid circles) agrees with the time sequence (empty squares) accurately solved for from the nonlinear ODE (13). The dynamics starts at $\psi _{0}=-0.6$, and its characteristics is significantly different from that of the time sequence in Fig. 2.

**Figure 4:**
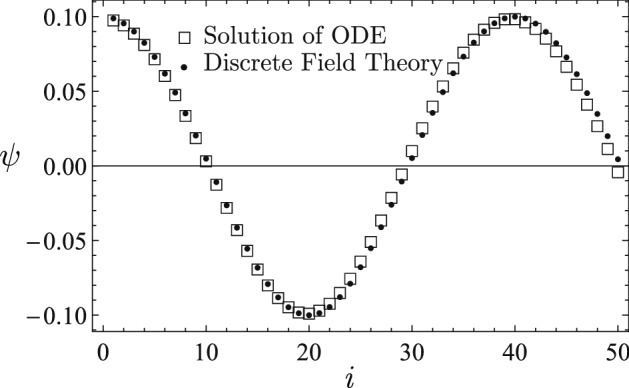
Starting at a much smaller amplitude, i.e., $\psi _{0}=0.1$, the predicted sequence (solid circles) shows the behavior of linear oscillation, in contrast with the strong nonlinearity of the sequence in Fig. 2 and the mild nonlinearity of the sequence in Fig. 3. The predicted time sequence agrees with the time sequence (empty squares) accurately solved for from the nonlinear ODE (13).

The learned discrete field theory is then served with two sets of new initial conditions, and the predicted time sequences are plotted using solid circle markers in Figs. 3 and 4 against the time sequences solved for from the nonlinear ODE (13). The predicted sequence in Fig. 3 starts at $\psi _{0}=-0.6$, and its dynamic characteristics is significantly different from that of the sequence in Fig. 2. The predicted sequence in Fig. 4 starts at a much smaller amplitude, i.e., $\psi _{0}=0.1$, and shows the behavior of linear oscillation, in contrast with the strong nonlinearity of the sequence in Fig. 2 and the mild nonlinearity of the sequence in Fig. 3. The agreement between the predictions of the learned discrete field theory and the accurate solutions of the nonlinear ODE (13) is satisfactory. These numerical results demonstrate that the proposed algorithms for machine learning and serving of discrete field theories are effective in terms of capturing the structure and predicting the dynamical behavior of the physical system.

### *Example 2*

**Figure 5:**
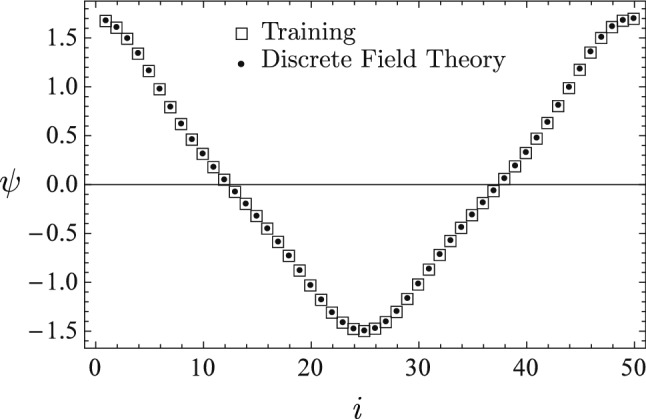
The training sequence (empty squares) represents a nonlinear oscillation in potential wall between $\psi =\pm 1.6$ in Fig. 6. The trained discrete Lagrangian density $L_{d}$ is accepted when the loss function $F({\overline{\psi }})$ on the training sequence is less than $10^{-7}$. The predicted sequence (solid circles) from the learned discrete field theory agrees very well with the training sequence.

**Figure 6:**
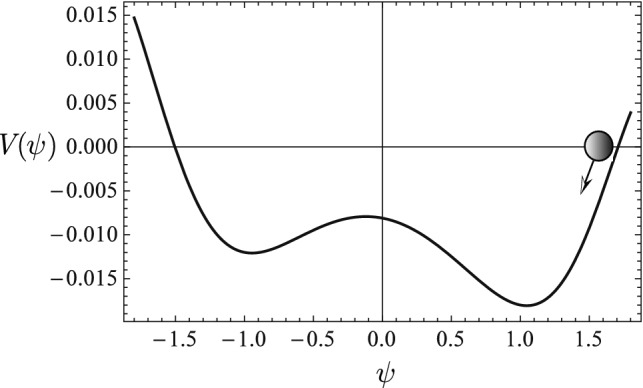
The training sequence in Fig. 5 represents a nonlinear oscillation in the large potential wall between $\psi =\pm 1.6$. There are two small potential wells secondary to the large potential well, one on the left between between $\psi =-1.3$ and $\psi =-0.1$, and one on the right between $\psi =-0.1$ and $\psi =1.5$.

The training data are plotted in Fig. 5 using empty square markers. It is a time sequence ${\bar{\psi }}_{i}$ $(i=0,...,50)$ generated by the nonlinear ODE

15 $$\begin{aligned} \psi ^{\prime \prime }-0.03\left[ \sin \left( 1-\psi ^{2}\right) \psi +0.1\right] =0 \end{aligned}$$

with initial conditions $\psi (t=0)=1.7$ and $\psi ^{\prime }(t=0)=0.$ The Lagrangian for the system is

16 $$\begin{aligned} L(\psi ,\psi ^{\prime })&=\frac{1}{2}\left( \psi ^{\prime }\right) ^{2}-V(\psi )\,,\end{aligned}$$

17 $$\begin{aligned} V(\psi )&=-0.015\left[ \cos \left( \psi ^{2}-1\right) +0.2\psi \right] \,, \end{aligned}$$

where $V(\psi )$ is a nonlinear potential plotted in Fig. 6. The training sequence represents a nonlinear oscillation in the potential well between $\psi =\pm 1.6$. The trained discrete Lagrangian density $L_{d}$ is accepted when the loss function $F({\overline{\psi }})$ on the training sequence is less than $10^{-7}$. The predicted sequence $\psi _{i}$ (solid circles in Fig. 5) by the serving algorithm from the learned discrete field theory agrees very well with the training sequence ${\bar{\psi }}_{i}$.

**Figure 7:**
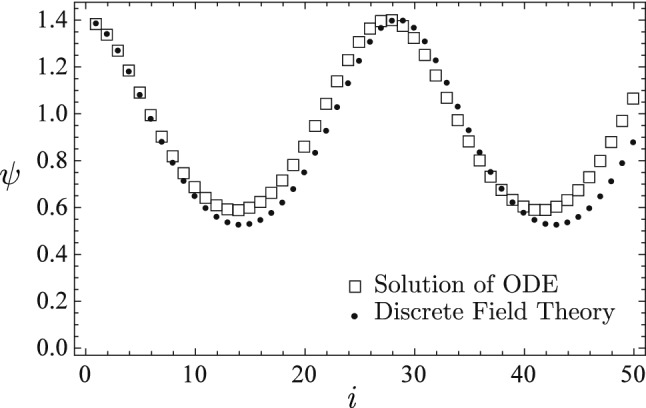
The learned discrete field theory correctly predicts a nonlinear oscillation in the small potential well between $\psi =-0.1$ and $\psi =1.5$ on the right of Fig. 6. The predicted sequence (solid circles) agrees with the accurate solution (empty squares) of the nonlinear ODE (15).

**Figure 8:**
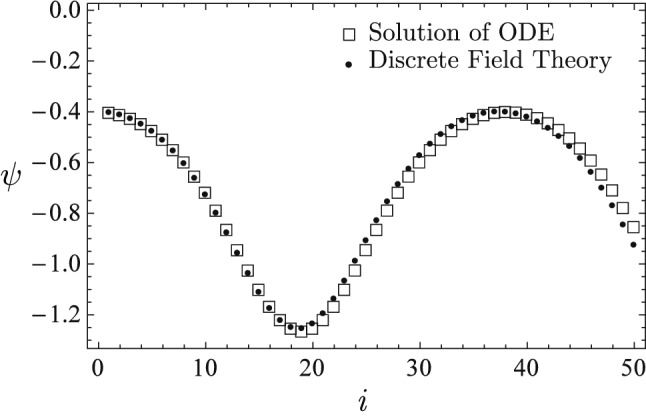
The learned discrete field theory correctly predicts a nonlinear oscillation in the small potential well between $\psi =-1.3$ and $\psi =-0.1$ on the left of Fig. 6. The predicted sequence (solid circles) agrees with the accurate solution (empty squares) of the nonlinear ODE (15).

The learned discrete field theory predicts two very different types of dynamical sequences shown in Figs. 7 and 8. The predicted sequences are plotted using solid circle markers and the sequences accurately solved for from the nonlinear ODE (15) are plotted using empty square markers. The sequence predicted in Fig. 7 is a nonlinear oscillation in the small potential well between $\psi =-0.1$ and $\psi =1.5$ on the right of Fig. 6, and the sequence predicted in Fig. 8 is a nonlinear oscillation in the small potential well between $\psi =-1.3$ and $\psi =-0.1$ on the left. For both cases, the predictions of the learned discrete field theory agree with the accurate solutions. Observe that in Fig. 6 the two small potential wells are secondary to the large potential wall between $\psi =\pm 1.6$. In Fig. 5 the small-scale fluctuations in the training sequence, which is a nonlinear oscillation in the large potential well, encode the structures of the small potential wells. The training algorithm is able to diagnose and record these fine structures in the learned discrete Lagrangian density, and the serving algorithm correctly predicts the secondary dynamics due to them.

## Kepler problem

**Figure 9:**
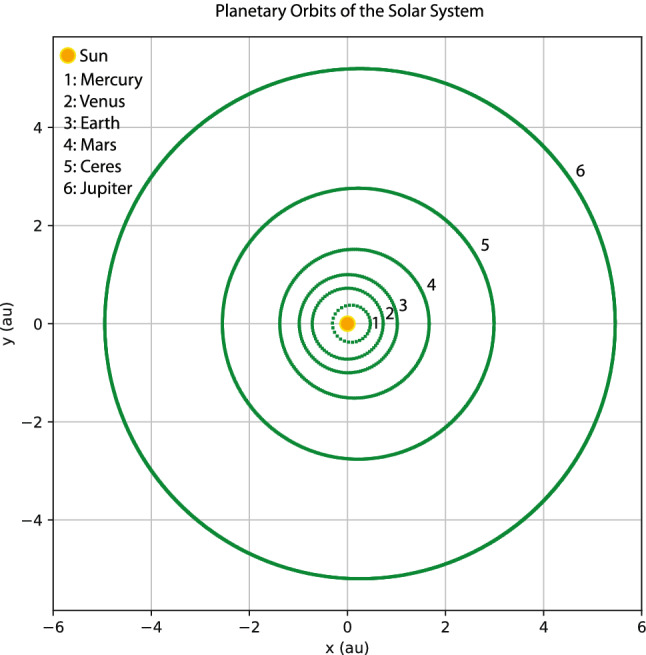
Orbits of the Mercury, Venus, Earth, Mars, Ceres and Jupiter generated by solving Newton’s equation of motion for a planet in the gravity field of the Sun according to Newton’s law of universal gravitation. These orbits are the training data for the discrete field theory.

In this section, to further demonstrate the effectiveness of the method developed, I apply it to the Kepler problem, which is concerned with dynamics of planetary orbits in the solar system. Let's turn the clock back to 1601, when Kepler inherited the observational data of planetary orbits meticulously collected by his mentor Tycho Brahe. It took Kepler 5 years to discover his first and second laws of planetary motion, and another 78 years before Newton solved the Kepler problem using his laws of motion and universal gravitation^84^. Assume that we have a set of data similar to that of Kepler, as displayed in Fig. 9. For simplicity, the data are the orbits of the Mercury, Venus, Earth, Mars, Ceres and Jupiter generated by solving Newton’s equation of motion for a planet in the gravity field of the Sun according to Newton’s law of universal gravitation. The spatial and temporal normalization scale-lengths are 1 a.u. and 58.14 days, respectively, and the time-steps of the orbital data is 0.05.

**Figure 10:**
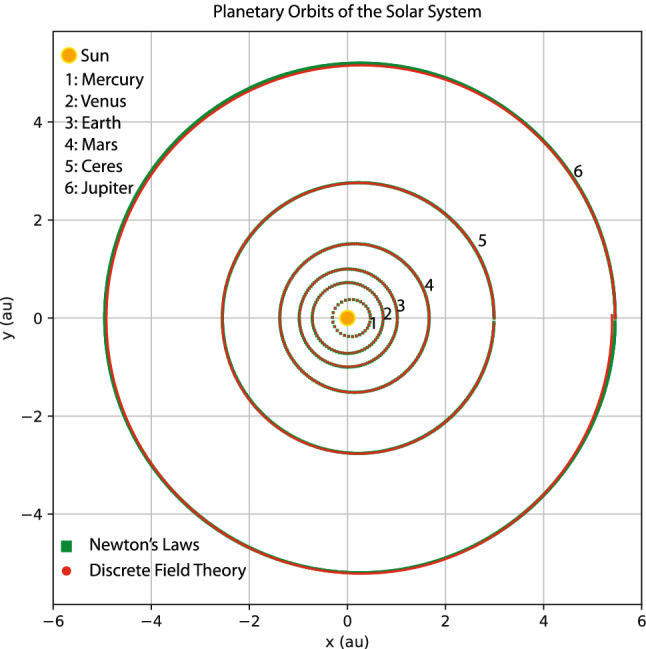
Orbits of the Mercury, Venus, Earth, Mars, Ceres and Jupiter. The orbits indicated by red markers are generated by the learned discrete field theory. The orbits indicated by green markers are the training orbits from Fig. 9.

My goal here is not to rediscover Kepler’s laws of planetary motion or Newton’s laws of motion and universal gravitation by machine learning. Instead, I train a discrete field theory from the orbits displayed in Fig. 9 and then serve it to predict new planetary orbits. For this case, the discrete field theory is about a 2D vector field defined on the time grid. Denote the field as $\psi _{i}=(x_{i},y_{i})$, where *i* is the index for the time grid, and $x_{i}$ and $y_{i}$ are the 2D coordinates of a planet in the solar system. In terms of the discrete field, the discrete Lagrangian density $L_{d}$ is a function on $\mathrm {R}^{4},$

18 $$\begin{aligned} L_{d}(\psi _{i},\psi _{i+1})=L_{d}(x_{i},y_{i},x_{i+1},y_{i+1})\,, \end{aligned}$$

and the DEL is a vector equation with two components,

19 $$\begin{aligned} EL_{x_{i}}&=\frac{\partial L_{d}(x_{i-1},y_{i-1},x_{i},y_{i})}{\partial x_{i}}+\frac{\partial L_{d}(x_{i},y_{i},x_{i+1},y_{i+1})}{\partial x_{i}}=0\,, \end{aligned}$$

20 $$\begin{aligned} EL_{y_{i}}&=\frac{\partial L_{d}(x_{i-1},y_{i-1},x_{i},y_{i})}{\partial y_{i}}+\frac{\partial L_{d}(x_{i},y_{i},x_{i+1},y_{i+1})}{\partial y_{i}}=0\,. \end{aligned}$$

The loss function on a data set $\psi =(x,y)$ is

$$\begin{aligned} F(x,y)=\frac{1}{I}\sum _{i=1}^{I-1}\left[ EL_{x_{i}}(x,y)^{2}+EL_{y_{i}}(x,y)^{2}\right] \,. \end{aligned}$$

Akin to the situation in “Examples of learning and predicting nonlinear oscillations” section, the serving algorithms preserves exactly an discrete symplectic form defined by

21 $$\begin{aligned} \Omega (x_{i},y_{i},x_{i+1},y_{i+1})=d\theta \,, \end{aligned}$$

22 $$\begin{aligned} \theta =\frac{\partial L_{d}(x_{i},y_{i},x_{i+1},y_{i+1})}{\partial x_{i+1}}dx_{i+1}+\frac{\partial L_{d}(x_{i},y_{i},x_{i+1},y_{i+1})}{\partial y_{i+1}}dy_{i+1}\,. \end{aligned}$$

**Figure 11:**
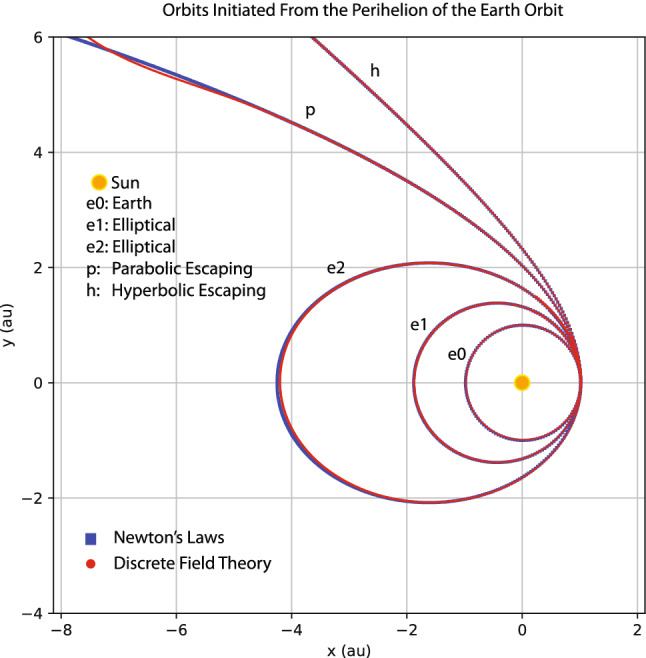
Orbits initiated from the Perihelion of the Earth orbit with initial velocities $v_{p}=0.98,1.13,1.26,1.40,\text { and }1.50.$ Orbit e0 is the Earth orbit. Orbits e1 and e2 are elliptical, and Orbit p is the parabolic escaping orbit and Orbit h is the hyperbolic escaping orbit. Red markers are the predictions of the trained discrete field theory, and blue markers are solutions according to Newton’s laws of motion and universal gravitation.

To model the discrete Lagrangian density $L_{d}(x_{i},y_{i},x_{i+1},y_{i+1})$, I use a fully connected neural network with two hidden layers, each of which has 40 neurons with the sigmoid activation function. The network is randomly initialized with a normal distribution, and then trained by the Adam optimizer^82^ until the averaged loss on a single time grid-point is reduced by a factor of $10^{5}$ relative to its initial value. Starting from the same initial conditions as the training orbits, the serving algorithm of the trained discrete field theory predicts the orbits plotted using red markers in Fig. 10 against the training orbits indicated by green markers. The agreement between the predicted and training orbits shown in the figure validates the discrete field theory learned. To serve it for the purpose of predicting new orbits, let’s consider the scenario of launching a device at the Perihelion of the Earth orbit with an orbital velocity $v_{p}$ larger than that of the Earth. Four such orbits, labeled by e1, e2, h, and p with $v_{p}=1.13,$ 1.26,  1.40,  and 1.50,  are plotted in Fig. 11 along with the orbit of the Earth, which is the inner most ellipse labeled by e0 with $v_{p}=0.98$. Orbits plotted using red markers are predictions of the trained discrete field theory, and orbits plotted using blue markers are solutions according to Newton’s laws of motion and gravitation. The agreement is satisfactory. Orbits e1 and e2 are elliptical, and Orbit p is the parabolic escaping orbit and Orbit h is the hyperbolic escaping orbit.

**Figure 12:**
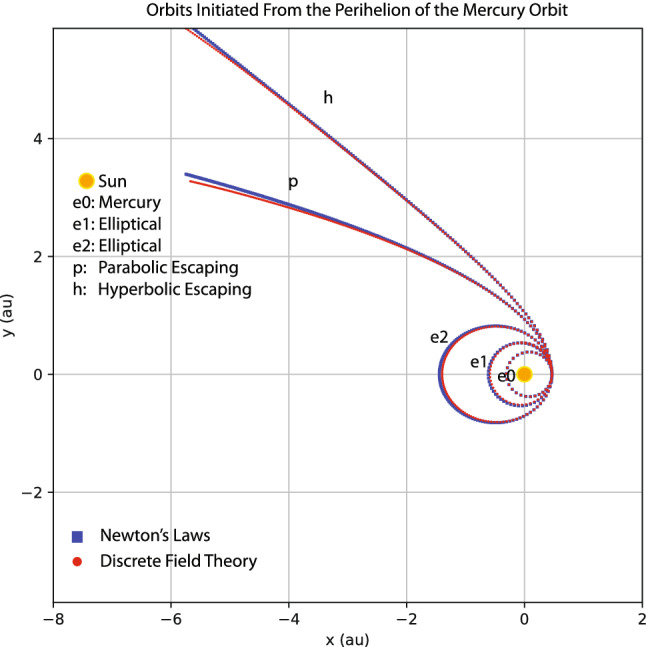
Orbits initiated from the Perihelion of the Mercury orbit with initial velocities $v_{p}=1.30,1.56,1.80,2.07,\text { and }2.20.$ Plotting markers and labels are similar to those in Fig. 11.

Similar study is carried out for the orbits initiated from the Perihelion of the Mercury orbit with an orbital velocities $v_{p}$ larger than that of the Mercury. Four such orbits are shown in Fig. 12 using the same plotting markers and labels as in Fig. 11. The inner most elliptical orbit is that of the Mercury with $v_{p}=1.30$. The orbit velocities at the Perihelion of the other four orbits are $v_{p}=1.56,$ 1.80, 2.07,  2.20. Again, the the predictions of the trained discrete field theory agree well with those of Newton’s laws.

It is noteworthy that the trained discrete field theory correctly predicts the parabolic and hyperbolic escaping orbits, even though the training orbits are all elliptical, see Figs. 9 and 10. Historically, Kepler argued that escaping orbits and elliptical orbits are governed by different laws. It was Newton who discovered or “learned” the 1/*r* dependency of the gravitational field from Kepler’s laws of planetary motion and Tycho Brahe’s data, and unified the elliptical orbits and escaping orbits under the same law of physics. Newton’s methodology has a deep and long-lasting influence on physicists after him. The results displayed in Figs. 11 and 12 show that the machine learning and serving algorithms solve the Kepler problem in terms of correctly prediction planetary orbits without knowing or learning Newton’s laws of motion and universal gravitation.

The algorithms developed are robust against variations of the governing laws of physics, because the method does not require any knowledge of the laws of physics other than the fundamental assumption that the governing laws are field theories. When the effects of special relativity or general relativity are important, the algorithms are expected to be valid as well.

**Figure 13:**
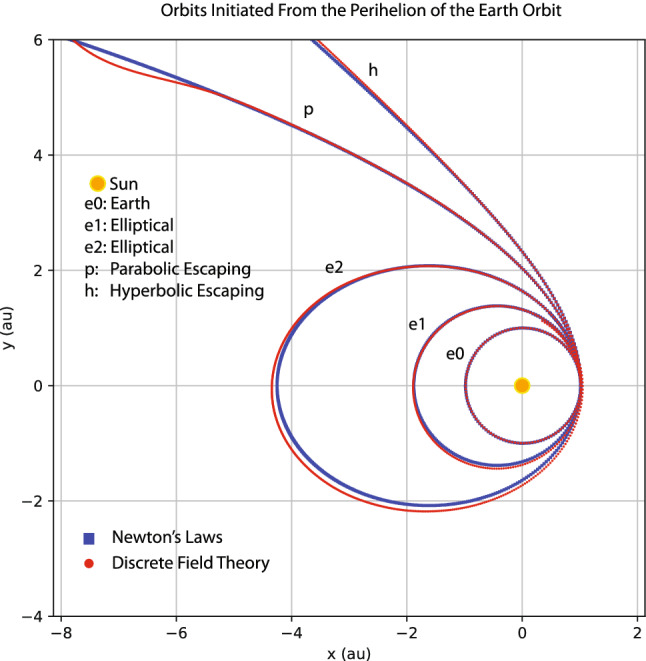
The effect of reduced training data. The orbit of the Ceres is removed from the training date shown in Fig. 9. The simulated orbits are the same as in Fig. 11. Red markers are the predictions of the trained discrete field theory, and blue markers are solutions according to Newton’s laws of motion and universal gravitation.

**Figure 14:**
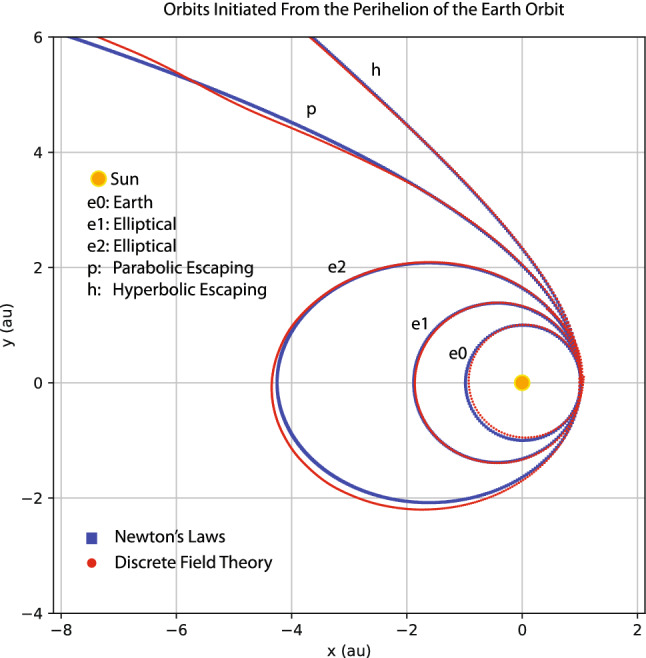
The effect of noise in training data. A Gaussian noise with zero mean and $10^{-3}$ standard deviation is added to the training data shown in Fig. 9. The simulated orbits are the same as in Fig. 11. Red markers are the predictions of the trained discrete field theory, and blue markers are solutions according to Newton’s laws of motion and universal gravitation.

Nevertheless, a few footnotes are in order. (i) There exist small discrepancies between the predictions from the learned discrete field theory and Newton’s laws in Figs. 11 and 12 when $r=\sqrt{x^{2}+y^{2}}\gtrsim 7$. This is because no training orbit in this domain is provided to the learning algorithm. The orbits predicted there are thus less accurate. To further understand the effect of reduced training data, I remove the orbit of the Ceres from the training data set and then apply the trained discrete Lagrangian to predict orbits initiated from the Perihelion of the Earth orbit as in Fig. 11. The result is displayed in Fig. 13, which shows a larger deviation from the prediction of Newton’s laws compared with Fig. 11. The increased discrepancies are understandable because, without the orbital data of the Ceres, there is no training data covering the space of $1.8\lesssim r\lesssim 4.5$. This indicates that trained discrete Lagrangian cannot accurately interpolate the gravitational field to regions without training data. On the other hand, without any additional physical assumption, there is no unique way to determine the gravitational field in a region without observational data. For example, this region could be vacuum or other small celestial bodies may exist and modify the gravitational field there. The neural network model of the discrete Lagrangian has the flexibility to learn the gravitational field in the region, whatever it might be, once training data in the region is available. But without the training data, the gravitational field in the region is under-determined in the neural network model. The calculation based on Newton’s laws implicitly assumes that the region is vacuum. Hence the larger discrepancies. (ii) The study presented above is meant to be a proof of principle. Practical factors, such as the noise of the training data and the three-body effects, are not included. Here, I would like to present a preliminary study on the effect of noise. In the orbital training data displayed in Fig. 9, I now add in a Gaussian noise with zero mean and $10^{-3}$ standard deviation. To denoise, the algorithm first feeds the data through a simple low-pass filter before training the discrete Lagrangian. The parameters of the filter are varied to minimize the loss function. The predictions of the trained discrete Lagrangian for the orbits initiated from the Perihelion of the Earth orbit are shown in Fig. 14. Comparing Figs. 11 and 14, we observe that the Gaussian noise at this level does not alter the predicted orbits substantially. However, the resulting error is not negligible and it is desirable to improve the robustness of the machine learning model against noise. We plan to carry out a systematic study on this topic. Several techniques are being considered. For instance, a noise-canceling signal can be searched to minimize the loss function during the training of the discrete Lagrangian. Generative machine learning models, which have found many applications in physics recently^23,41,43,85,86^, can be applied for the purpose of denoising as well^85,87^.

## Conclusions and discussion

In this paper, a method for machine learning and serving of discrete field theories in physics is developed. The learning algorithm trains a discrete field theory from a set of observational data of the field on a spacetime lattice, and the serving algorithm employs the learned discrete field theory to predict new observations of the field for given new boundary and initial conditions.

The algorithm does not attempt to capture statistical properties of the training data, nor does it try to discover differential equations that govern the training data. Instead, it learns a discrete field theory that underpins the observed field. Because the learned field theory is discrete, it overcomes the difficulties associated with the learning of continuous theories. Compared with continuous field theories, discrete field theories can be served more easily and with improved long-term accuracy and fidelity. The serving algorithm of discrete field theories belongs to the family of structure-preserving geometric algorithms^46–75^, which have been proven to be superior to the conventional algorithms based on discretization of differential equations. The demonstrated advantages of discrete field theories relative to continuous theories in terms of machine learning compatibility are consistent with Bostrom’s simulation hypothesis^78^.

I should point out that in general, a specific machine learning algorithm is often more effective for certain types of data. The data relevant to the present study are assumed to be observations of physical fields in spacetime governed by field theories, even though laws of physics in specific forms, such as Newton’s laws of motion and gravity, are not needed for the algorithms developed in the present study to be effective in terms of correctly predicting observations. Admittedly, the assumption of the existence of an underpinning field theory puts a strong constraint on the applicability of the method. Many physics and engineering problems do not have field theoretical formulations. Most non-conservative systems fall into this category. The algorithms developed in the present study certainly do not apply in these situations. Further investigation is needed to incorporate non-conservative effects.

In addition, the machine learning community has developed large amounts of innovative methods and techniques, many of which can be explored to facilitate the machine learning of discrete field theories. For instance, if a data set is generated by systems governed by different Lagrangians, we can include both the observations and the corresponding discrete Lagrangians as the training data for a generative model^23,41,43,85,86^ using the method of variational autoencoder^88^. After training, the encoder establishes a map between observations and discrete Lagrangians. And the space of discrete Lagrangian can be continuously sampled to generate new Lagrangians and predict new observations. The generative model can also be utilized to remove noise in the training data, as discussed in “Kepler problem” section.

We plan to pursue these topics in the follow-up study.
